# Association between obstructive sleep apnea syndrome and metabolic syndrome: a severity-stratified meta-analysis

**DOI:** 10.3389/fmed.2026.1850790

**Published:** 2026-05-14

**Authors:** Dasheng Chen, Xing Zhang, Changchuan Liu, Yuanjun Li, Wei Hu

**Affiliations:** 1 Yan’an University, Yan’an, Shaanxi, China; 2 Jintang County Second People's Hospital, Sichuan, China; 3 Department of Respiratory Medicine, Affiliated Hospital of Yan'an University, Yan’an, Shaanxi, China

**Keywords:** hyperglycemia, hypertension, meta-analysis, MetS, OSAS

## Abstract

**Objective:**

This study aimed to systematically investigate the association between obstructive sleep apnea syndrome (OSAS) and metabolic syndrome (MetS), with a particular focus on severity–stratified analysis, in order to clarify the differential risks of MetS and its components (hypertension, hyperglycemia, etc. ) among patients with different levels of OSAS severity.

**Methods:**

A comprehensive literature search was conducted in Web of Science, PubMed, Cochrane, and Embase databases from their inception to June 1, 2025, using keywords related to “sleep apnea syndrome” and “MetS”. After rigorous quality evaluation and data extraction of the included studies, a stratified meta-analysis by OSAS severity was performed using Stata 17.0 software.Subgroup analysis and meta-regression were further applied to explore heterogeneity sources, enhancing the reliability of results.

**Results:**

A total of 10 studies involving 10,205 participants were included. The meta-analysis revealed that the risk of developing MetS in patients with moderate-to-severe OSAS was 2.18 times higher than in those with mild OSAS (OR = 2.18, 95%CI:1.30–3.68, *P* < 0.001). The risk of hypertension in patients with moderate-to-severe OSAS was 2.19 times higher than that in patients with mild OSAS (OR = 2.19, 95%CI:1.57–3.06, *P* < 0.05). The risk of hyperglycemia in patients with moderate-to-severe OSAS was 1.50 times higher than that in patients with mild OSAS(OR = 1.50, 95%CI:1.01–2.18). Subgroup analysis results showed that heterogeneity existed between studies published before 2016 (*I*^2^ = 54.6%, *P* = 0.051) and those published in or after 2016 (*I*^2^ = 65.2%, *P* = 0.035). Meta-regression analysis indicated that the heterogeneity in the results of studies on the association between OSAS and the risk of MetS was primarily due to the year of publication of the literature (2016).

**Conclusion:**

This study found that patients with moderate to severe OSAS have a 2.18 times higher risk of developing MetS than those with mild OSAS. Their risk of hypertension and hyperglycemia also goes up significantly. For patients with moderate to severe OSAS, it makes sense to prioritize MetS screening and start early intervention to lower their risk of developing MetS.

**Systematic Review Registration:**

https://www.crd.york.ac.uk/PROSPERO/.

## 1 Background

OSAS is a common public health disorder. Approximately 936 million adults aged 30–69 worldwide are affected by it, among whom 425 million have moderate to severe cases. The characteristic of this disorder is the repeated obstruction of the upper airway during sleep, which leads to hypoxia, hypercapnia and deterioration of sleep quality, and is associated with various diseases such as cardiovascular and endocrine disorders (1–3). There is also a risk of cognitive impairment and accelerated aging (4). MetS is a disease characterized by a series of complex physiological, biochemical, and metabolic abnormalities (5). The global prevalence of MetS in adults is estimated to be approximately 30% (6). There are many differences in the definition of “MetS”, but the diagnosis mainly includes hypertension, obesity, high triglyceride levels, low high-density lipoprotein levels, and Hyperglycemia levels (7–9). Patients with sleep apnea syndrome are 6 to 9 times more likely to develop MetS than the general population (10). MetS and OSAS can both cause damage to multiple systems, including the cardiovascular, endocrine, and central nervous systems, with the most severe damage occurring in the cardiovascular system. Previous meta-analyses have focused on the risk of MetS in the general population and in OSAS populations, but there has been a lack of stratified studies in OSAS populations and a lack of exploration of the sources of heterogeneity (11, 12). This study introduces a severity-stratified approach, which was lacking in previous meta-analyses, to compare MetS risk between moderate-to-severe and mild OSAS, while also exploring heterogeneity sources. The goal is to alert clinicians to the metabolic indicators of patients with moderate-to-severe OSAS and ultimately reduce the incidence of MetS. This study has been registered with PROSPERO (CRD420251064866).

## 2 Data and methods

### 2.1 Data

Relevant literature on sleep apnea OSAS and MetS was retrieved from English literature databases (Embase, PubMed, Corchene, Web of Science). The search was conducted from the date of database establishment to June 1, 2025. References from relevant reviews and selected studies were further searched.

#### 2.1.1 Inclusion criteria

(1) Population: Patients diagnosed with OSAS according to established guidelines. (2) Exposure: Moderate-to-severe OSAS (AHI≥15) or mild OSAS (AHI < 15). (3) Comparison: For the severity-stratified analysis, the comparison group was mild OSAS, whereas for the overall analysis, non-OSAS participants were used as the control. (4) Outcome: Metabolic syndrome (MetS) and its components (e.g., hypertension, hyperglycemia, etc.). (5) Study type: Case-control studies, cohort studies, cross-sectional studies.

#### 2.1.2 Exclusion criteria

(1) Literature with a score of less than 6 on the Newcastle-Ottawa Scale (NOS) or the Agency for Healthcare Research and Quality (AHRQ) scale. (2) Inconsistent research objectives/data could not be extracted. (3) Duplicate publications. (4) Literature for which the full text could not be obtained even after contacting the authors. (5) Literature review, conference papers, animal studies, and case reports. (6)Unable to download.

### 2.2 Method

#### 2.2.1 Search strategy

A combination of subject terms and free-text search terms is employed. The following terms are used: sleep apnea syndrome, sleep/sleep apnea/sleep hypoventilation/periodic breathing with somnolence/mixed central and obstructive sleep apnea/mixed central and obstructive sleep apnea/mixed sleep apnea/breathing, sleep disorders/sleep-related breathing disorders/ MetS/Levin syndrome X/MetS X/cardiovascular MetS/MetS X, insulin resistance/metabolic X syndrome/metabolic X syndrome, metabolic disorder/cardiovascular MetS/cardiovascular MetS as English search terms. The English search formula is illustrated using PubMed as an example. See Table 1.

**Table 1:** PubMed search strategy.

Number	Search terms
#7	#3 AND #6
#6	#5 OR #4
#5	((((((((Metabolic Syndromes[Title/Abstract]) OR (Reaven Syndrome X[Title/Abstract])) OR (Metabolic Syndrome X[Title/Abstract])) OR (Cardiovascular Syndrome, Metabolic[Title/Abstract])) OR (Syndrome X, Insulin Resistance[Title/Abstract])) OR (Metabolic X Syndrome[Title/Abstract])) OR (Syndrome X, Dysmetabolic[Title/Abstract])) OR (Cardiometabolic Syndromes[Title/Abstract])) OR (Syndrome, Cardiometabolic[Title/Abstract])
#4	“Metabolic Syndrome”[Mesh]
#3	#2 OR #1
#2	(“Sleep Apnea Syndromes”[Mesh]) OR ((((((((((Apnea Syndrome, Sleep[Title/Abstract]) OR (Sleep Apnea[Title/Abstract])) OR (Sleep Hypopnea[Title/Abstract])) OR (Hypersomnia with Periodic Respiration[Title/Abstract])) OR (Sleep Apnea, Mixed Central and Obstructive[Title/Abstract])) OR (Mixed Central and Obstructive Sleep Apnea[Title/Abstract])) OR (Sleep Apnea, Mixed[Title/Abstract])) OR (Mixed Sleep Apneas[Title/Abstract])) OR (Breathing, Sleep-Disordered[Title/Abstract])) OR (Sleep Disordered Breathing[Title/Abstract]))
#1	“Sleep Apnea Syndromes”[Mesh]

#### 2.2.2 OSAS diagnostic criteria

OSAS diagnosis requires patients to exhibit symptoms of sleep-related breathing disorders (snoring, gasping, or apnea), excessive daytime sleepiness or fatigue despite adequate sleep opportunities, which cannot be explained by other medical conditions; and five or more respiratory events per hour of sleep, primarily obstructive respiratory events (obstructive or mixed apnea, hypoventilation, or RERA) (AHI ≥5). OSAS can also be diagnosed in asymptomatic cases with an AHI ≥ 15 events per hour. The severity criteria are as follows: mild: AHI ≥5 and < 15 events per hour; moderate: ≥15 events per hour to < 30 events per hour; severe: ≥ 30 events per hour (13). In this study, moderate-to-severe OSAS was defined as an AHI ≥ 15 events per hour.

#### 2.2.3 Outcome measures

Original literature should provide detailed data on the study population, and known data can be used to calculate odds ratios (OR) and 95% confidence intervals (CI) as outcome measures.

#### 2.2.4 Literature screening

Two researchers who have received systematic evidence-based training independently screened the literature according to the inclusion and exclusion criteria, extracted data, and cross-checked the results. In case of disagreement, they discussed the issue or consulted external experts for assistance in making a decision. All retrieved literature was imported into NoteExpress to remove duplicate documents. The titles and abstracts of the literature were read for initial screening, and literature clearly unrelated to the theme of this study was excluded. The full text was then read again to screen the literature, and the final literature to be included was determined. Literature information was extracted using Excel software, including first author, publication date, study region, study type, study population, and influencing factors.

#### 2.2.5 Literature quality assessment

Cohort studies and case-control studies were assessed for quality using the NOS (14). The NOS scale includes three categories: study population selection, comparability between groups, and exposure or outcome assessment, with a total of eight evaluation criteria. The maximum score is 9 points, with 0–3 points indicating low quality, 4–6 points indicating moderate quality, and 7 points or higher indicating high-quality literature.

Cross-sectional studies use the AHRQ criteria, which include 11 criteria, with a maximum score of 11 points. Responses are given as “yes,” “no,” or “unknown.” Scores of 0–4 indicate low-quality literature, 5–7 indicate moderate-quality literature, and 8 or higher indicate high-quality literature (15).

#### 2.2.6 Statistic alanalysis

Data were analyzed using Stata 17.0 software for meta-analysis. The odds ratio (OR) was used as the combined effect size, and its 95% confidence interval was calculated. The Q test was employed for heterogeneity analysis, and the *I*^2^ statistic was used to assess the degree of heterogeneity among study results. The results of the heterogeneity test were combined to determine whether to select a fixed-effects model or a random-effects model. If *P* > 0.1 and I^2^ < 50%, the included studies were considered homogeneous, and the fixed-effects model was used; if *P* ≤ 0.1 and *I*^2^ ≥ 50%, the included studies were considered heterogeneous, and the random-effects model was selected. Sensitivity analysis and subgroup analysis were conducted on studies with potential heterogeneity to explore the sources of heterogeneity, and meta-regression analysis was further performed to determine the sources of heterogeneity in the results. Funnel plots and Egger linear regression are used to assess publication bias in the included studies.

## 3 Research results

### 3.1 Literature search results

**Figure 1:**
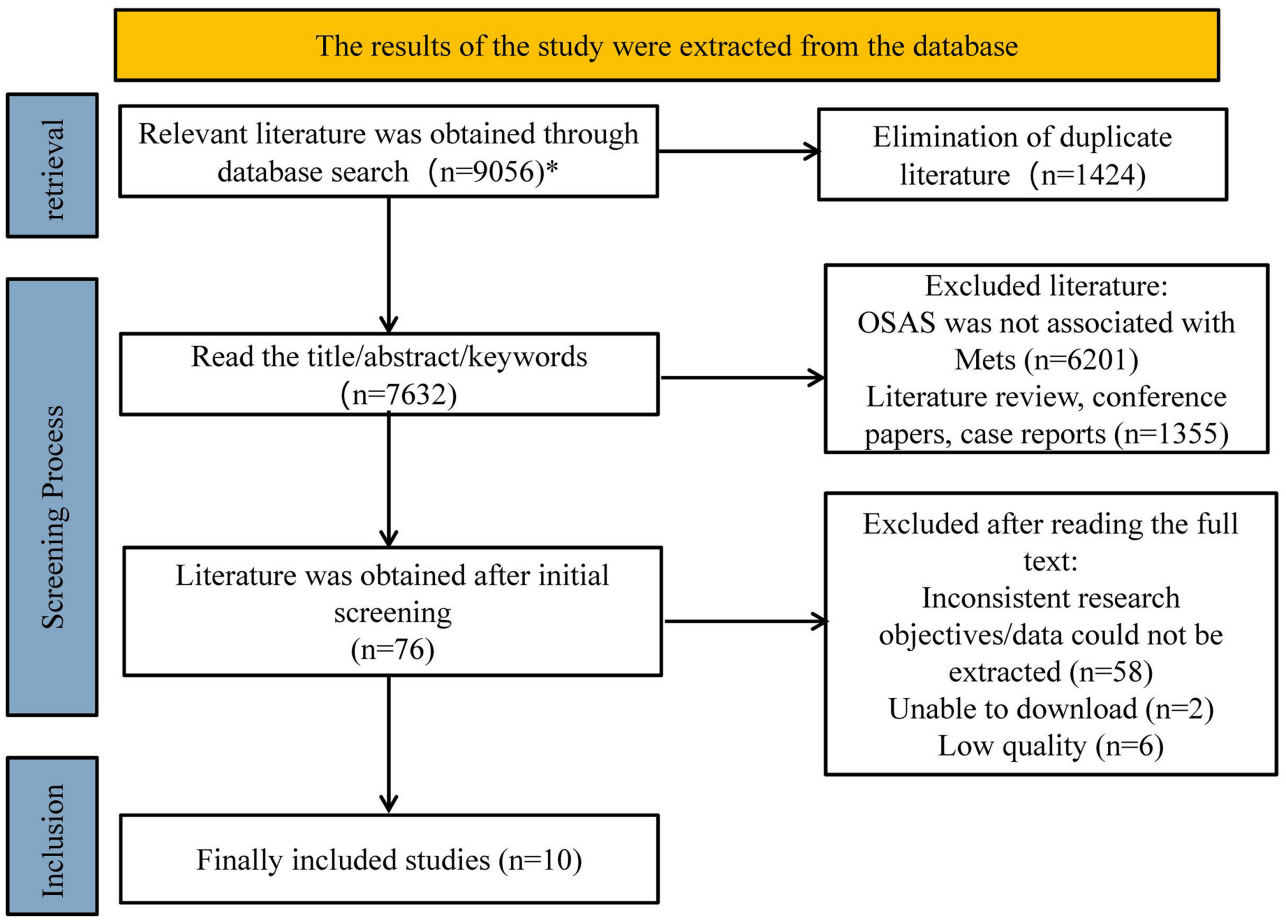
Literature search process ^*^ databases searched and the literature detected are as follows: PupMeded (*n* = 631), Embase (*n* = 5283), Cochrane (*n* = 314), Web of Science (*n* = 2828).

Following strict literature search, screening, and evaluation methods, a total of 9,056 documents were initially obtained through the search strategy. After removing 1,424 duplicate documents from various databases, 6,201 articles were excluded after reviewing the titles, abstracts, and keywords for irrelevance to the topic, 1,355 articles were excluded as literature reviews, case reports, or conference papers, 58 articles were excluded after reading the full text for mismatched research objectives or inability to extract data, 2 articles were excluded for inability to download, and 6 articles were excluded for low scores. Ultimately, 10 studies were included in the meta-analysis, including 4 case-control studies and 6 cross-sectional studies. See Figure 1.

### 3.2 Basic evaluation and quality assessment of included studies

The total sample size of this study was 10,205 cases, among which 4,923 cases were in the OSAS group and 5,282 cases were in the non-OSAS group. In the OSAS group, 1,853 patients had MetS, and 664 patients had MetS in the non-OSAS group. In the studies that could be classified into OSAS groups, 1,756 cases were moderate to severe OSAS, 581 cases were mild OSAS. The case-control studies using the NOS scale had a quality score of greater than 6, and the cross-sectional studies using the AHRQ scale had a quality score of greater than 6. The basic information of the included literature can be seen in Table 2.

**Table 2:** Basic characteristics and quality evaluation results of the included literature.

Author	Year	Type	Male (%)	MetS (%)	Degree of OSAS		OSAS (%)	MetS diag	Population	Quality score
					Mild (%)	Moderate-severe (%)				
Wu et al. (36)	2015	CST	245 (100.00)	139 (56.73)	–	–	161 (65.71)	I	Asia	6
Ozol et al. (37)	2011	CST	90 (72.00)	27 (21.6)	27 (25.71)	78 (74.29)	105 (84.00)	II	Asia	6
Agrawal et al. (38)	2011	CST	178 (78.41)	167 (73.56)	41 (21.92)	146 (78.08)	187 (82.37)	II	Asia	6
Coughlin et al. (39)	2004	CCT	104 (100.0)	68 (65.38)	–	–	61 (58.65)	II	Non–Asia	7
Muraki et al. (40)	2010	CST	1710 (37.02)	776 (16.84)	–	–	1217 (26.42)	II	Asia	7
Sasanabe et al. (41)	2006	CST	778 (87.79)	403 (44.38)	179 (21.85)	640 (78.15)	819 (90.19)	I	Asia	7
Chen et al. (42)	2016	CCT	312 (75.91)	238 (57.9)	67 (18.55)	294 (81.45)	361 (87.83)	II	Asia	7
Tseng et al. (43)	2017	CCT	99 (82.50)	36 (30.00)	–	–	40 (33.33)	II	Asia	7
Fernando et al. (44)	2024	CCT	700 (68.00)	553 (52.17)	267 (30.86)	598 (69.14)	865 (83.98)	II	Non–Asia	6
Deepali et al. (45)	2025	CST	1297 (51.10)	110 (4.60)	–	–	1107 (45.57)	I	Non–Asia	8

CCT, Case-control; CST, Cross-sectional. 2. population: Non-Asia include: Europe, South America, North America. 3. MetS Diagnosis:I:IDF/Other national guidelines and consensus.; II:NCEP-ATPIII. 4.-: Representative no data.

### 3.3 Meta-analysis results for the risk of developing MetS

#### 3.3.1 Risk of MetS components in moderate-to-severe OSAS

**Figure 2:**
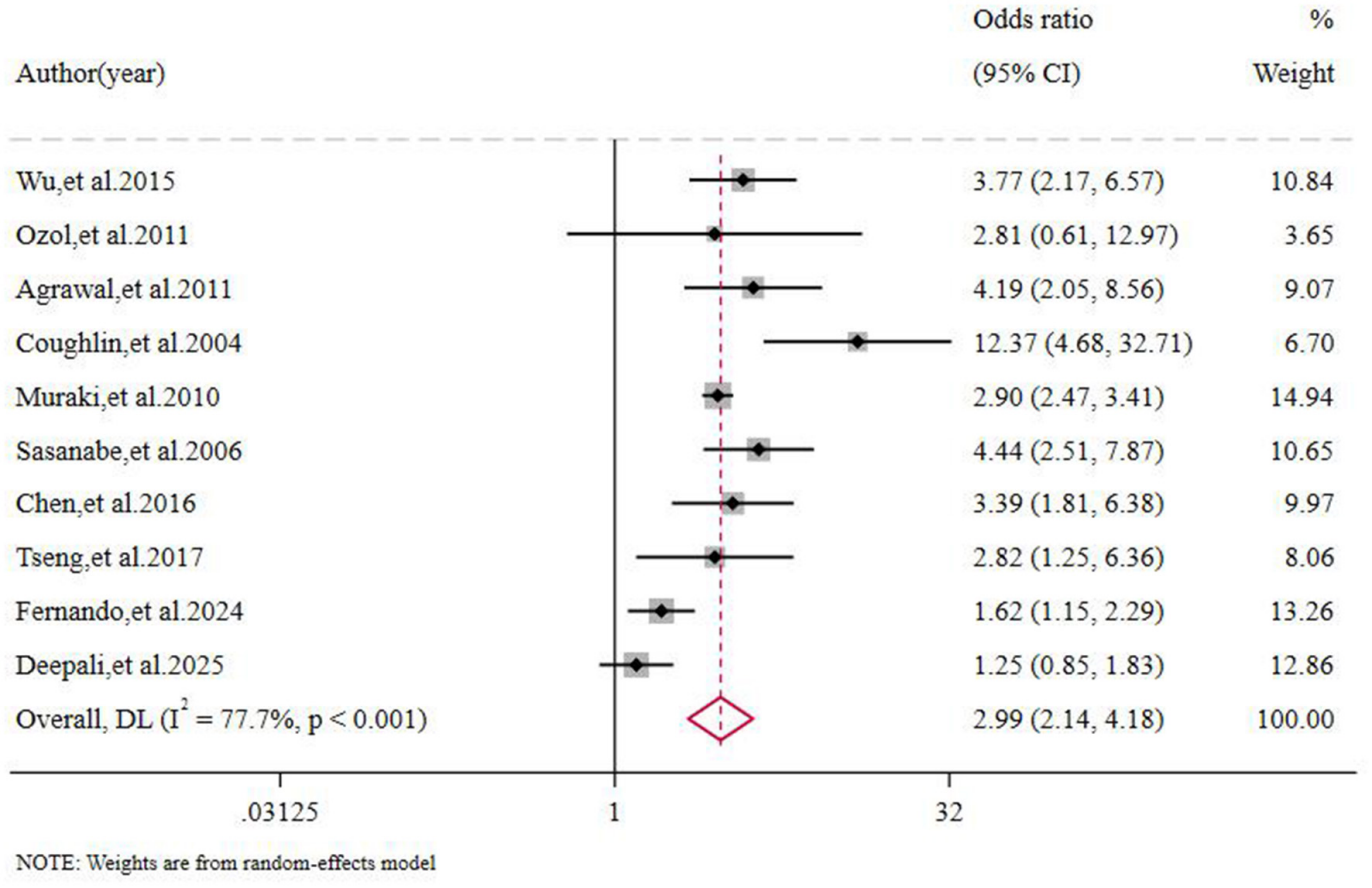
Forest plot of the association between OSAS patients and MetS risk.

There was significant variability in the results of studies examining the association between OSAS and the risk of MetS (*I*^2^ = 77.7%, *P* < 0.001). A random-effects model was used to conduct a meta-analysis of the effect sizes of the included studies. The results (study sample sizes are listed in Table 2) showed that the risk of developing MetS in OSAS patients was 2.99 times higher than in the non-OSAS population (O = 2.99, 95% CI: 2.14–4.18, *P* < 0.001), with the difference being statistically significant Figure 2.

**Figure 3:**
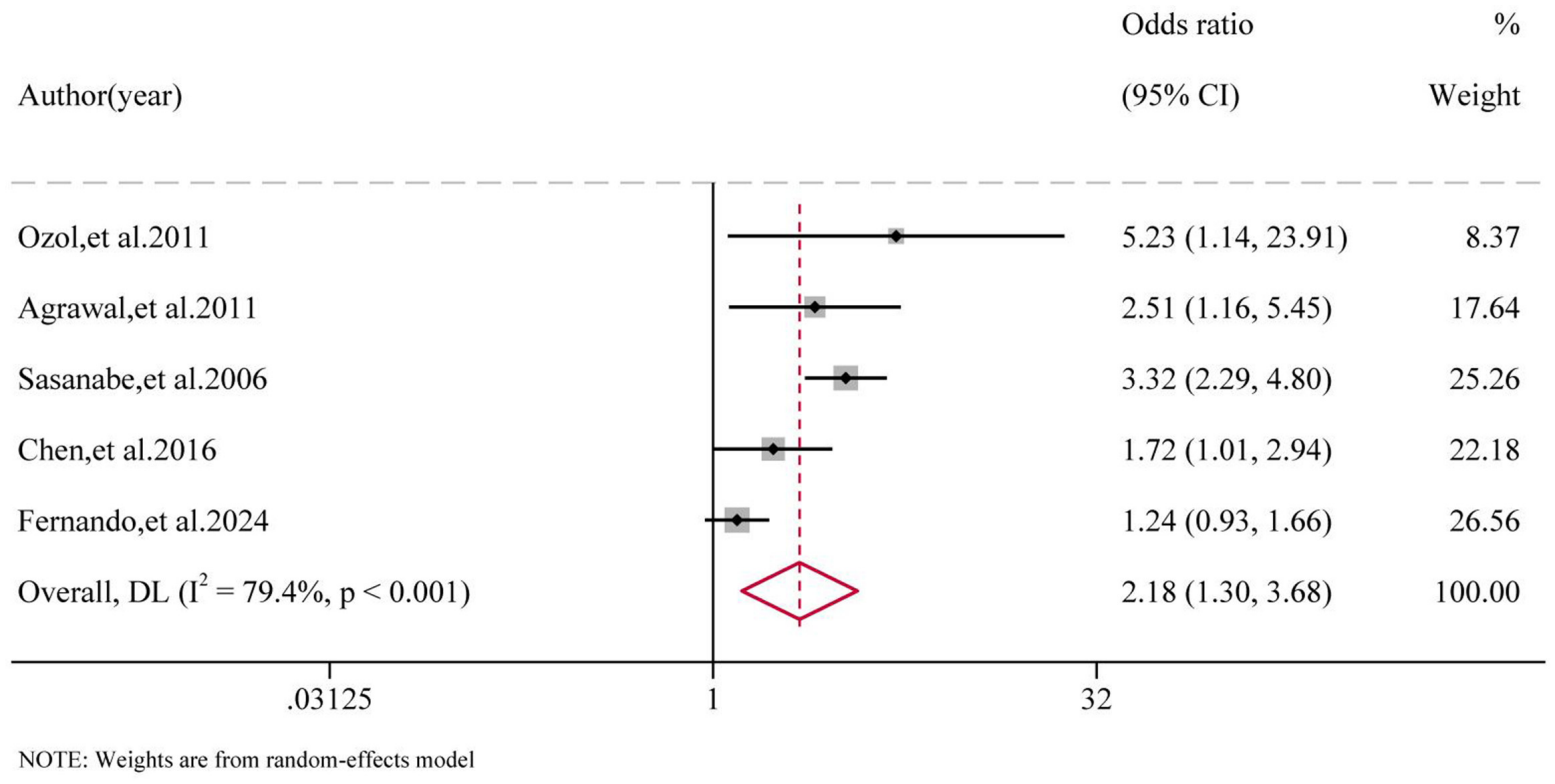
Forest plot of moderate-to-severe OSAS patients and MetS risk.

The risk of developing MetS in patients with moderate to severe OSAS is 2.18 times higher than in patients with mild OSAS (OR = 2.18, 95%CI: 1.30–3.68, *P* < 0.001). See Figure 3.

The risk of developing hypertension in patients with moderate-to-severe OSAS is 2.19 times higher than in patients with mild OSAS (OR = 2.19, 95%CI:1.57–3.06, *P* < 0.05). The risk of developing Hyperglycemia in patients with moderate-to-severe OSAS is 1.50 times higher than in patients with mild OSAS (OR = 1.50, 95%CI:1.01–2.18, *P* < 0.05). The risk of developing hyperlipidemia and central obesity in patients with moderate-to-severe OSAS is not significantly different from that in patients with mild OSAS (*P* < 0.05). For details, please refer to Table 3.

**Table 3:** Association between moderate-to-severe OSAS and components of MetS.

Variable	Moderate–severe OSAS		Mild OSAS		All *OR*	95%CI	All I^2^(%)	Z	*P*
	Events	Total	Events	Total					
**Hyperglycemia**					1.50	1.01–2.18	47.00%	2.07	< 0.05
Ozol et al.	15	78	4	27					
Sasanabe et al.	167	640	23	179					
Chen et al.	104	294	21	67					
Fernando et al.	165	598	63	267					
**Hypertension**					2.19	1.57–3.06	52.90%	4.65	< 0.001
Ozol et al.	35	78	6	27					
Sasanabe et al.	458	640	84	179					
Chen et al.	223	294	40	67					
Fernando et al.	353	598	125	267					
**Hyperlipidemia**					1.20	0.7–2.15	83.40%	0.67	>0.05
Sasanabe et al.	432	640	103	179					
Chen et al.	148	294	25	67					
Fernando et al.	217	598	88	267					
**Central obesity**					1.42	0.19–10.60	98.20%	0.30	>0.05
Sasanabe et al.	541	640	117	179					
Chen et al.	248	294	54	67					
Fernando et al.	502	598	201	267					

#### 3.3.2 Subgroup analysis results

Subgroup analysis: year of publication, study design type, MetS Diagnosis, population, proportion of males, and total sample size. Analysis by study design type revealed heterogeneity between studies in both cross-sectional studies (*I*^2^ = 77.3%, *P* < 0.01) and case-control studies (*I*^2^ = 82.5%, *P* < 0.01). Therefore, the random-effects model was used to pool the effect sizes. When analyzing by MetS Diagnosis, heterogeneity was observed between studies with diagnosis method *I* (*I*^2^ = 88.8%, *P* < 0.01) and diagnosis method II (*I*^2^ = 70.4%, *P* = 0.002), and the random-effects model was also used to pool the effect sizes. When analyzing by publication year, there was heterogeneity between studies published before 2016 (*I*^2^ = 54.6%, *P* = 0.051) and those published in or after 2016 (*I*^2^ = 65.2%, *P* = 0.035). When analyzing by the proportion of males in the studies, when the proportion of males was >70%, the risk of developing MetS increased by 3.35 times, with heterogeneity (*I*^2^ = 7.8%, *P* = 0.369). When the proportion of males was ≤ 70%, there was heterogeneity (*I*^2^ = 90.7%, *P* < 0.01), and a random-effects model was used to pool the effect sizes. Analysis was also conducted based on population and total sample, both of which showed heterogeneity. The specific pooled effect sizes and their 95% confidence intervals are presented in Table 4.

**Table 4:** Subgroup risk of MetS in OSAS vs non–OSAS patients.

Subgroup	Pooled effect size			Heterogeneity		
	Number of literatures	*OR*	95%CI	I^2^ (%)	*P*	EM
Type of study						
CCT	4	3.42	1.59–7.39	82.5	< 0.001	R
CST	6	2.89	1.9–4.36	77.3	< 0.001	R
Population						
Asia	7	3.09	2.69–3.55	0.00	0.747	F
Non–Asia	3	2.51	1.07–5.88	89.2	< 0.001	R
MetS Diagnois						
I	3	2.70	1.14–6.37	88.8	0.1	R
II	7	3.15	2.16–4.60	70.4	< 0.001	R
Year						
< 2016	6	4.01	2.81–5.71	54.6	0.051	R
≥2016	4	1.92	1.26–2.93	65.2	0.035	R
Male (%)						
< 70%	3	1.84	1.06–3.20	90.7	< 0.001	R
≥70%	7	4.11	3.09–5.45	7.80	0.369	F
Sample						
< 900	7	3.45	2.12–5.60	71.6	0.002	R
≥900	3	2.48	1.32–4.65	89.6	< 0.001	R

#### 3.3.3 Meta-regression analysis results

Meta-regression analysis was further conducted to explore the potential sources of heterogeneity among subgroups. Based on the results of subgroup analysis and the publication year of the papers, the following variables were included in the regression equation: publication year, study population, proportion of males, sample size, study design type, and method of diagnosing MetS. The results of the meta-regression analysis indicated that the publication year of the literature may be a source of heterogeneity between groups. The *P*–values for other variables were >0.05. The specific results are shown in Table 5.

**Table 5:** Meta regression analysis of OSAS and risk of MetS.

variate	β	SE	t	*P*	95%CI
Type of study	−3.812	0.256	−1.93	0.149	−10.094–2.469
Year	−4.922	0.385	−3.28	0.046	−0.628–1.021
population	3.994	1.542	2.59	0.081	−0.913–8.902
MetS diagnois	−0.734	1.514	−0.46	0.682	−5.873–4.403
Proportion of males	−4.027	1.920	−2.10	0.127	−10.139–2.084
Sample	2.446	1.572	1.56	0.218	−2.557–7.449

#### 3.3.4 Sensitivity and publication bias

**Figure 4:**
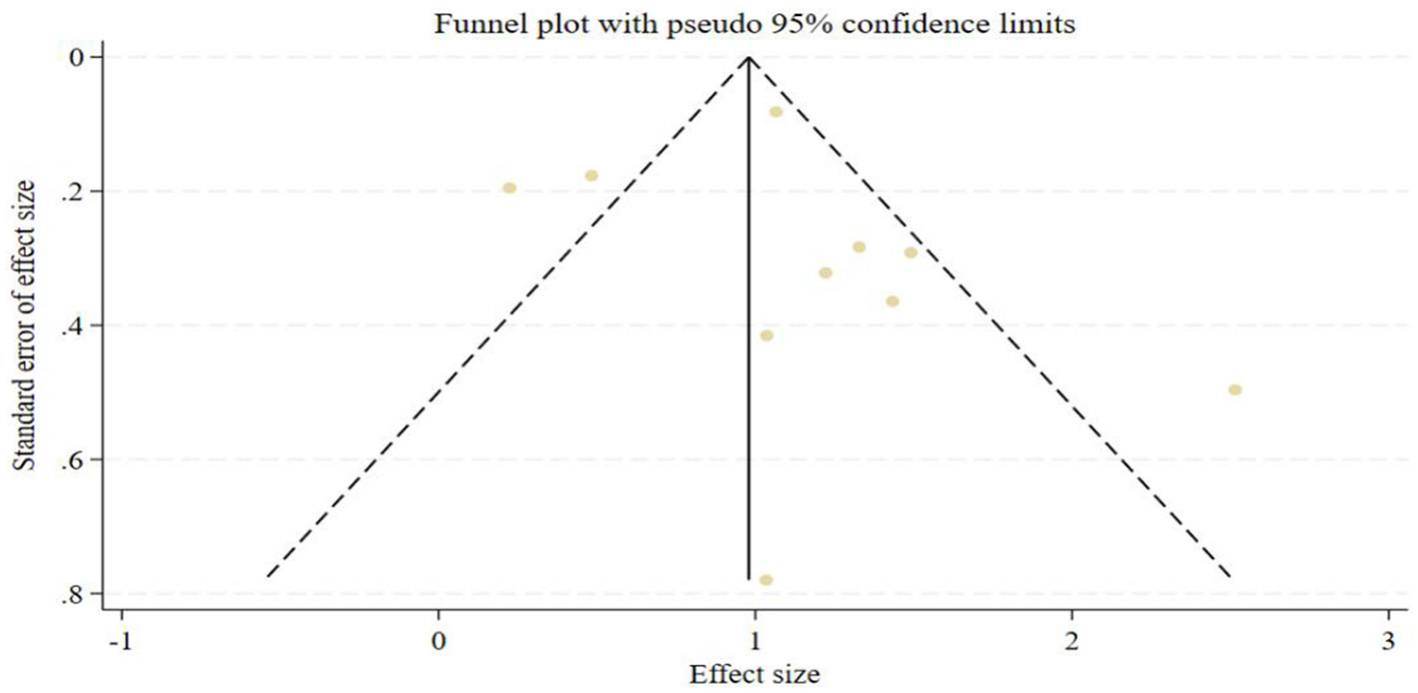
Funnel plot of literature publication bias for OSAS and risk of MetS.

Sensitivity analysis was conducted using the stepwise exclusion method to assess the stability of the results from the 10 included studies. The results indicated that the weights assigned to each included study had no significant impact on the overall analysis results. This study employed funnel plots, Begg's test, and Egger's linear regression analysis to assess whether the pooled effect size was subject to publication bias. Egger's regression analysis showed (*t* = 0.66, *P* = 0.526), Begg's test results (*z* = 0.18, *P* = 0.858), and the funnel plot was generally symmetrical on both sides, roughly forming an inverted funnel shape. In summary, the meta-analysis results did not show publication bias. The funnel plot is shown in Figure 4.

## 4 Discussion

The association between OSAS and MetS has been extensively investigated. Studies have found a positive association between the two conditions (16). The findings of Jacob et al. (17) indicate that the risk of developing MetS in patients with OSAS is 4.94 times higher than in those without OSAS (95% CI: 3.68–6.65), consistent with the results of other studies (12).

This meta analysis confirmed the association between OSAS and MetS, showing that OSAS increases the risk of MetS. It also identified publication year (2016) as a source of heterogeneity. Studies conducted in 2016 or later indicated that the risk of developing MetS among OSAS patients was 1.92 times higher than that of non-OSAS patients (95%CI:1.26–2.93). Studies published in or after 2016 showed that OSAS patients had a 1.92-fold higher risk of developing MetS compared to non-OSAS patients (95% CI: 1.26–2.93). This change may be attributed to the rapid advancement of medical technology in recent years and increased health awareness among the general population. For example, the development of portable and continuous monitoring devices has provided a convenient, cost-effective, and efficient option for diagnosing OSAS (18). Additionally, predictive models for the occurrence of MetS have demonstrated good predictive capability (19). These measures promote early screening and awareness of diseases, enabling timely and effective intervention and treatment.

Subgroup analysis revealed that Asian patients with OSAS had a higher risk of MetS than patients from other regions (specifically, South America, Oceania, and Europe). This finding is inconsistent with the results of some earlier studies (20, 21). From a genetic perspective, genome-wide association studies (GWAS) in the Han Chinese population have identified OSA risk loci (such as PACRG and SLC52A3) (22), which are primarily associated with intermediate phenotypes related to OSA, such as obesity and sleep structure disorders. These loci may indirectly influence OSA susceptibility by regulating energy metabolism and neural function. The heritability of MetS is approximately 10–30% (23). The genetic evidence cited in this study is based on single-population GWAS; future cross-racial cohort studies are needed to validate the universality of these loci. The “metabolically obese normal-weight” phenotype in Asian populations (i.e., visceral fat accumulation in the abdomen and insulin resistance despite normal BMI) and the “thrifty genotype” (genes that store energy during periods of food instability) may exacerbate the risk of MetS during nutritional transitions (24). MetS in Asian populations is not only influenced by genetic factors but is also closely related to environmental factors, such as high-salt diets and lack of physical activity in some regions, which are also contributing factors (25).

Studies have found that male patients with OSAS have a higher risk of developing MetS than females (26), which is consistent with the results of this study. From a sociodemographic perspective, male OSAS patients have higher rates of alcohol consumption and smoking, which may exacerbate insulin resistance (27), In severe OSA patients who smoke, the risk of dyslipidemia and insulin resistance increases by 1.34-fold and 4.06-fold, respectively (28). From the perspective of adipocyte biology, male visceral adipocytes exhibit larger volumes and higher lipolytic activity, thereby releasing more free fatty acids (FFAs), which can lead to insulin resistance and increased triglyceride synthesis (29).

Patients with moderate-to-severe OSAS (AHI ≥ 15) experience more frequent apnea events and more severe intermittent hypoxemia. A study by Hou et al. (30) indicated that patients with moderate-to-severe OSAS have a higher risk of hypertension than those with mild OSAS, which is consistent with the findings of the present study. Patients with moderate-to-severe OSAS suffer from chronic hypoxia and more severe apneic episodes. This hypoxia activates carotid body chemoreceptors, triggering sympathetic nervous system activation and the release of catecholamines (e.g., norepinephrine), which raise blood pressure. Additionally, this activation can stimulate the renin-angiotensin-aldosterone system (RAAS), promoting vasoconstriction and increased aldosterone secretion, which contributes to the development of hypertension (31, 32).

Aronsohn et al. (33) further noted that the severity of OSAS is significantly associated with the risk of diabetes. Patients with moderate-to-severe OSAS had a glycated hemoglobin increase approximately three times that of patients with mild OSAS, similar to the findings of this study. The pathogenesis of glucose metabolism disorders in OSAS patients is multifactorial. One key mechanism is that chronic hypoxia enhances sympathetic nervous system excitability, which in turn promotes glucose dysregulation. Additionally, intermittent hypoxia can cause reduced insulin sensitivity and impaired pancreatic beta cell function, thereby triggering insulin resistance (34, 35).

No statistically significant association was found between severe OSAS and hyperlipidemia or central obesity compared to mild OSAS (*P* > 0.05). Heterogeneity was high, which may relate to differences in how studies defined indicators such as waist circumference for central obesity. Small sample sizes may also have limited statistical power. Thus, the current data do not confirm an association, nor do they rule out clinical relevance. This study has some limitations. It relies mainly on cross-sectional designs, so causal relationships cannot be firmly established. It also included only English-language publications, which could introduce language restrictions. However, the NOS/AHRQ scores reflect study design and internal validity and are not influenced by language. Despite these limitations, the stratified design and heterogeneity analysis offer new insights for mechanistic research on metabolic risks associated with OSAS. Future prospective cohort studies are essential to clarify causality between moderate-to-severe OSAS and MetS and to examine differences in mechanisms across populations

## 5 Conclusion

This study demonstrates a positive correlation between OSAS and the risk of developing MetS. Patients with moderate to severe OSAS have a significantly higher risk of MetS than those with mild OSAS, providing evidence to support stratified management. Regular monitoring of blood glucose and blood pressure may be considered for these patients.

## Data Availability

The original contributions presented in the study are included in the article/supplementary material, further inquiries can be directed to the corresponding authors.
