# Correction: Association between obstructive sleep apnea syndrome and metabolic syndrome: a severity-stratified meta-analysis

**DOI:** 10.3389/fmed.2026.1894213

**Published:** 2026-06-24

**Authors:** Dasheng Chen, Xing Zhang, Changchuan Liu, Yuanjun Li, Wei Hu

**Affiliations:** 1 Yan'an University, Yan'an, Shaanxi, China; 2 Jintang County Second People's Hospital, Sichuan, China; 3 Department of Respiratory Medicine, Affiliated Hospital of Yan'an University, Yan'an, Shaanxi, China

**Keywords:** hyperglycemia, hypertension, meta-analysis, MetS, OSAS

There was a mistake in Table 3 as published. The total sample sizes (the “Total” columns) for several studies were incorrect. 1. Hyperglycemia: Ozol et al., Moderate-Severe OSAS total: originally 63; Sasanabe et al., Moderate-Severe OSAS total: originally 473; Chen et al., Moderate-Severe OSAS total: originally 190; Fernando et al., Moderate-Severe OSAS total: originally 433. 2. Hypertension: Ozol et al., Moderate-Severe OSAS total: originally 113; Sasanabe et al., Moderate-Severe OSAS total: originally 1098; Chen et al., Moderate-Severe OSAS total: originally 517; Fernando et al., Moderate-Severe OSAS total: originally 951; Ozol et al., Mild OSAS total: originally 28. 3.Hyperlipidemia: Fernando et al., Moderate-Severe OSAS total: originally 815. 4. Central obesity: Fernando et al., Moderate-Severe OSAS total: originally 815; Chen et al., Mild OSAS total: originally 121. The corrected Table 3 appears below.

**Table 3:** Association between moderate-to-severe OSAS and components of MetS.

Variable	Moderate-severe OSAS		Mild OSAS		All *OR*	95% CI	All I^2^ (%)	Z	*P*
	Events	Total	Events	Total					
**Hyperglycemia**					1.50	1.01–2.18	47.00%	2.07	< 0.05
Ozol et al.	15	78	4	27					
Sasanabe et al.	167	640	23	179					
Chen et al.	104	294	21	67					
Fernando et al.	165	598	63	267					
**Hypertension**					2.19	1.57–3.06	52.90%	4.65	< 0.001
Ozol et al.	35	78	6	27					
Sasanabe et al.	458	640	84	179					
Chen et al.	223	294	40	67					
Fernando et al.	353	598	125	267					
**Hyperlipidemia**					1.20	0.7–2.15	83.40%	0.67	>0.05
Sasanabe et al.	432	640	103	179					
Chen et al.	148	294	25	67					
Fernando et al.	217	598	88	267					
**Central obesity**					1.42	0.19–10.60	98.20%	0.30	>0.05
Sasanabe et al.	541	640	117	179					
Chen et al.	248	294	54	67					
Fernando et al.	502	598	201	267					

The original version of this article has been updated.

